# CIP2A is a target of bortezomib in human triple negative breast cancer cells

**DOI:** 10.1186/bcr3175

**Published:** 2012-04-26

**Authors:** Ling-Ming Tseng, Chun-Yu Liu, Kung-Chi Chang, Pei-Yi Chu, Chung-Wai Shiau, Kuen-Feng Chen

**Affiliations:** 1 Department of Surgery, Taipei Veterans General Hospital, No. 201 Sec. 2 Shih-Pai Road, Taipei 112, Taiwan; 2 Institute of Biopharmaceutical Sciences, National Yang-Ming University, No. 155 Sec. 2 Li-Nong Street, Taipei 112, Taiwan; 3 School of Medicine, National Yang-Ming University, No. 155 Sec. 2 Li-Nong Street, Taipei 112, Taiwan; 4 Division of Hematology and Oncology, Department of Medicine, Taipei Veterans General Hospital, No. 201 Sec. 2 Shih-Pai Road, Taipei 112, Taiwan; 5 Department of Pathology, St. Martin De Porres Hospital, No. 565 Sec. 2 Daya Road, Chiayi 600, Taiwan; 6 Department of Medical Research, National Taiwan University Hospital, No. 7 Chung-Shan S Road, Taipei 100, Taipei, Taiwan; 7 National Center of Excellence for Clinical Trial and Research, National Taiwan University Hospital, No. 7 Chung-Shan S Road, Taipei 100, Taiwan

## Abstract

**Introduction:**

Triple negative breast cancer (TNBC) is very aggressive and currently has no specific therapeutic targets, such as hormone receptors or human epidermal growth factor receptor type 2 (HER2); therefore, prognosis is poor. Bortezomib, a proteasome inhibitor, may exert efficacy in TNBC through its multiple cellular effects. Here, we tested the efficacy of bortezomib and examined the drug mechanism in breast cancer cells.

**Methods:**

Five breast cancer cell lines: TNBC HCC-1937, MDA-MB-231, and MDA-MB-468; HER2-overexpressing MDA-MB-453; and estrogen receptor positive MCF-7 were used for *in vitro *studies. Apoptosis was examined by both flow cytometry and Western Blot. Signal transduction pathways in cells were assessed by Western Blot. Gene silencing was done by small interfering RNA (siRNA). *In vivo *efficacy of bortezomib was tested in nude mice with breast cancer xenografts. Immunohistochemical study was performed on tumor tissues from patients with TNBC.

**Results:**

Bortezomib induced significant apoptosis, which was independent of its proteasome inhibition, in the three TNBC cell lines, but not in MDA-MB-453 or MCF-7 cells. Furthermore, cancerous inhibitor of protein phosphatase 2A (CIP2A), a cellular inhibitor of protein phosphatase 2A (PP2A), mediated the apoptotic effect of bortezomib. We showed that bortezomib inhibited CIP2A in association with p-Akt downregulation in a dose- and time-dependent manner in all sensitive TNBC cells, whereas no alterations in CIP2A expression and p-Akt were noted in bortezomib-resistant cells. Overexpression of CIP2A upregulated p-Akt and protected MDA-MB-231 and MDA-MB-468 cells from bortezomib-induced apoptosis, whereas silencing CIP2A by siRNA overcame the resistance to bortezomib-induced apoptosis in MCF-7 cells. In addition, bortezomib downregulated CIP2A mRNA but did not affect the degradation of CIP2A protein. Furthermore, bortezomib exerted *in vivo *antitumor activity in HCC-1937 xenografted tumors, but not in MCF-7 tumors. Bortezomib downregulated CIP2A expression in the HCC-1937 tumors but not in the MCF-7 tumors. Importantly, CIP2A expression is readily detectable in tumor samples from TNBC patients.

**Conclusions:**

CIP2A is a major determinant mediating bortezomib-induced apoptosis in TNBC cells. CIP2A may thus be a potential therapeutic target in TNBC.

## Introduction

Triple negative breast cancer (TNBC), which comprises approximately 15% of all breast carcinomas [1], is defined as breast carcinoma that does not express estrogen receptor (ER), progesterone receptor (PgR) or human epidermal growth factor receptor type 2 (HER2). These tumors are characterized by occurrence in younger women, aggressive behaviors with a high recurrence rate, metastasis potential and poor prognosis [1-3]. Because of a lack of targeted therapies (such as hormone therapy or anti-HER2 therapy) for TNBC, chemotherapy is currently the main treatment. There is, therefore, an urgent and unmet need to develop targeted therapy for TNBC. Discovering the critical molecular mechanisms of TNBC and developing new compounds targeting these mechanisms may advance the development of TNBC treatments.

Bortezomib is the first proteasome inhibitor to be approved for treatment for multiple myeloma and mantle cell lymphoma [4,5]. Bortezomib has been shown to block proteasome degradation of IκB, an inhibitor of nuclear factor-κB (NF-κB), and demonstrated remarkable anti-tumor activity against these hematological malignancies. The transcription factor NF-κB is believed to play a vital role in the action of bortezomib as it is involved in cancer cell proliferation, apoptosis, invasion, metastasis, tumorigenesis and angiogenesis [4-6]. In addition, bortezomib affects several other cellular pathways, such as tumor suppressor protein p53, cell cycle regulators p21, p27, proapoptotic (Noxa, bax, and so on) and antiapoptotic (mcl-1, bcl-2, and so on) bcl-2 family proteins that lead to apoptosis [7]. Preclinical studies have demonstrated an *in vitro *antitumor effect of bortezomib in breast cancer models [8-10]. In the clinical arena bortezomib as a single agent showed limited clinical efficacy (objective response) in two single institutional phase II clinical trials for patients with previously treated metastatic breast cancers (MBC) [11,12]. In contrast, combinational trials of bortezomib with other therapeutics for MBC seem promising: a phase II study combining bortezomib with pegylated liposomal doxorubicin demonstrated a response rate of 8% in patients with MBC [13]; another phase I/II study showed that a combination of bortezomib and capecitabine is well tolerated and has moderate antitumor activity (15% overall response rate) in heavily pretreated MBC patients [14]; and another phase I/II study combining bortezomib with docetaxel showed a more promising response rate of 38% at the maximum tolerated dose for anthracycline-pretreated advanced/metastatic breast cancer [15]. Bortezomib is currently being tested in combination with fulvestrant, a novel estrogen antagonist, in a randomized phase II study for patients with ER positive MBC (NCT01142401). Although the reason why the single bortezomib regimen is not significantly active in clinical trials might be explained by the possibility of the activation of multiple drug resistance pathways in heavily pretreated populations, particularly those previously exposed to anthracycline [16], alternative mechanisms might also confer sensitivity to bortezomib in patients with breast cancers. Interestingly, in the phase II study by Yang *et al. *[12], the inhibition of proteasome activity was measured in bortezomib-treated patients and did not translate into a meaningful therapeutic benefit in these patients, implying that bortezomib's mechanism of action may not necessarily depend on its proteasome inhibitory effect [12]. Therefore, the exact anti-tumor mechanisms of bortezomib in breast cancers, and to our interest TNBC, warrant further elucidation.

In this regard, our previous study showed that downregulation of phospho-Akt (p-Akt) plays a key role in determining the sensitivity of hepatocellular carcinoma (HCC) cells to bortezomib-induced apoptosis [17]. Importantly, we found that the differential cytotoxic effects of bortezomib on HCC are independent of its proteasome inhibition [17]. Akt is a well-known key player in cancer cell survival and apoptosis regulation. It is noteworthy that activated p-Akt signaling has been shown to be higher in TNBC tumor samples than in other breast tumor types [18]. Negative regulation of Akt signaling can be achieved by phosphatases, such as phosphatase and tensin homologue deleted on chromosome ten (PTEN), and protein phosphatase 2A (PP2A). PTEN dephosphorylates phosphatidylinositol 3, 4, 5-triphosphate (PIP3) at the 3-position to counteract phosphatidylinositol-3-kinase (PI3K), thereby inhibiting Akt signaling. In contrast, PP2A is a complex serine/threonine protein phosphatase that can directly dephosphorylate oncogenic kinases such as p-Akt and p-ERK [19], by which PP2A can function as a tumor suppressor through regulating apoptosis, cell cycle, cell survival and proliferation [20]. Our recent data indicated that the bortezomib enhances PP2A activity thereby downregulating p-Akt and inducing apoptosis in HCC cells [21]. We also found that bortezomib can act synergistically with sorafenib to induce apoptosis in HCC cells through this PP2A-dependent p-Akt inactivation [22]]. In addition, several cellular upstream inhibitors of PP2A such as SET [23], and cancerous inhibitor of protein phosphatase 2A (CIP2A) [24] have been identified. SET is a nucleus/cytoplasm-localized phosphoprotein that has been shown to be predominantly a myeloid leukemia-associated protein [25]. In contrast, CIP2A has emerged as a novel oncoprotein and a growing number of reports have shown its overexpression in many human malignancies, including breast cancers [21,24,26-31]. CIP2A has been shown to promote anchorage-independent cell growth and *in vivo *tumor formation by inhibiting PP2A activity toward c-Myc [32]. Importantly, Come *et al. *[33] found that CIP2A is associated with clinical aggressiveness in human breast cancer and promotes the malignant growth of breast cancer cells, suggesting CIP2A as a new target for breast cancer therapy.

In this study, we revealed that CIP2A, a cellular inhibitor of protein phosphatase 2A (PP2A), mediated the apoptotic effect of bortezomib. Bortezomib induced significant apoptosis in TNBC cell lines but not in hormone receptor positive or HER2-overexpressing cells. Our data indicate that bortezomib's downregulation of CIP2A and p-Akt correlated with its drug sensitivity. Through ectopic overexpression and silencing CIP2A, we confirmed that CIP2A is the predominant mediator of bortezomib-induced apoptosis in TNBC cells. This CIP2A-dependent p-Akt inhibitory mechanism that mediates the efficacy of bortezomib was confirmed *in vivo *in a nude mouse model. Furthermore, CIP2A expression can be demonstrated in tumor samples from TNBC patients. Our results suggest that CIP2A may be a novel therapeutic target for treatment of TNBC.

## Materials and methods

### Reagents and antibodies

Bortezomib (Velcade^®^) was kindly provided by Millennium Pharmaceuticals (Cambridge, MA, USA)For *in vitro *studies, bortezomib at various concentrations was dissolved in dimethyl sulfoxide (DMSO) and then added to cells in (D)MEM medium (Invitrogen, Carlsbad, CA, USA). The final DMSO concentration was 0.1% after addition to the medium. Antibodies for immunoblotting such as anti-I-κB and CIP2A were purchased from Santa Cruz Biotechnology (San Diego, CA, USA). Other antibodies such as anti-caspase-3, Akt, and P-Akt (Ser473) were from Cell Signaling (Danvers, MA, USA).

### Cell culture and western blot analysis

The HCC-1937, MDA-MB-231, MDA-MB-468, MDA-MB-453 and MCF-7 cell lines were obtained from American Type Culture Collection (Manassas, VA, USA). All breast cancer cells were maintained in (D)MEM medium supplemented with 10% FBS, 0.1 mM nonessential amino acids (NEAA), 2 mM L-glutamine, 100 units/mL penicillin G, 100 μg/mL streptomycin sulfate, and 25 μg/mL amphotericin B in a 37°C humidified incubator and an atmosphere of 5% CO_2 _in air. Lysates of breast cancer cells treated with drugs at the indicated concentrations for various periods of time were prepared for immunoblotting of caspase-3, P-Akt, Akt, CIP2A, and so on. Western blot analysis was performed as previously reported [17].

### Apoptosis analysis

Drug-induced apoptotic cell death was assessed using the following methods: (a) Western blot analysis of caspase activation or poly ADP-ribose polymerase (PARP) cleavage (in caspase deficient MCF-7 cells), and (b) measurement of apoptotic cells by flow cytometry (sub-G1 analysis).

### Proteasome inhibitory activity

A 20S Proteasome Activity Assay kit (Chemicon International, Temecula, CA, USA) was used to determine the proteasome inhibition in drug-treated cells. All procedures were conducted according to the manufacturer's instructions [17]. In brief, cells were treated with or without bortezomib for the indicated length of time. Then, cells were lysed and total protein was quantified. Equal amounts of total protein of each sample were used for incubation with the proteasome substrate (fluorophore-labeled substrate). Proteasome activity measurement was based on detection of the fluorophore after cleavage from the labeled substrate by a fluorometer with a 380/460 nm filter set.

### Gene knockdown using siRNA

Smartpool siRNA reagents, including control (D-001810-01), and CIP2A were all purchased from Dharmacon (Chicago, IL, USA). The procedure has been described previously [17]. Briefly, cells were transfected with siRNA (final concentration, 100 nM) in six-well plates using the Dharma-FECT1 transfection reagent (Dharmacon) according to the manufacturer's instructions. After 72 hours, the medium was replaced and the breast cancer cells were incubated with bortezomib, harvested, and separated for western blot analysis and for apoptosis analysis by flow cytometry.

### Generation of MDA-MB-231 and MDA-MB-468 cells with constitutive active CIP2A

Cells were transfected with active CIP2A construct by procedures as previously described [26]. Briefly, following transfection, cells were incubated in the presence of G418 (1.40 mg/mL). After eight weeks of selection, surviving colonies, that is, those arising from stably transfected cells, were selected and individually amplified. CIP2A cDNA (KIAA1524) was purchased from Origene (RC219918; Rockville, MD, USA) and constructed into pCMV6 vector.

### Xenograft tumor growth

NCr athymic nude mice (five to seven weeks of age) were obtained from the National Laboratory Animal Center (Taipei, Taiwan). The mice were housed in groups and maintained in a specific pathogen free (SPF)-environment. All experimental procedures using these mice were performed in accordance with protocols approved by the Institutional Animal Care and Use Committee of Taipei Veterans General Hospital. Each mouse was inoculated s.c. in the dorsal flank with 2 to 4 × 10^6 ^breast cancer cells suspended in 0.1 to 0.2 mL serum-free medium containing 50% Matrigel (BD Biosciences, Bedford, MA, USA) under isoflurane anesthesia. Tumors were measured using calipers and their volumes calculated using a standard formula: width^2 ^× length × 0.52. When tumors reached 100 mm^3^, mice were administered an i.p. injection of bortezomib (0.5 mg/kg body weight) twice weekly for three to four weeks. Controls received vehicle.

### Reverse transcription-PCR

Total RNA was extracted from cultured cells using TRIzol^® ^Reagent (Invitrogen, San Diego, CA, USA) and RT-PCR was performed according to the manufacturer's instructions (MBI Fermentas, Vilnius, Lithuania). RT-PCR analyses were performed as previously described, using specific primers for human CIP2A (forward, 5'-TGGCAAGATTGACCTGGGATTTGGA-3'; reverse, 5'-AGGAGTAATCAAACGTGGGTCCTGA-3'; 172 bps); the GAPDH (glyceraldehyde-3-phosphate dehydrogenase) gene (forward, 5'-CGACCACTTTGTCAAGCTCA-3'; reverse, 5'-AGGGGTCTACAT GGCAACTG-3'; 228 bps) was chosen as an internal control.

Real-time quantitative PCR was performed in a LightCycler 480II instrument (Roche Diagnostics, Indianapolis, IN, USA) using a LightCycler 480 SYBR Green I Master kit (Roche). Primers are the same as above described.

### Immunohistochemical staining

Paraffin-embedded tissue microarray of tumor samples from TNBC patients' sections (4-μm) on poly-1-lysine-coated slides were first de-waxed in xylene and re-hydrated through graded alcohols, followed by a rinse using 10 mM Tris-HCl (pH 7.4) and 150 mM sodium chloride, then treated with 3% hydrogen peroxide for five minutes. Slides were incubated with a 1:100 dilution of rabbit polyclonal anti-p90 autoantigen (CIP2A) antibody (ab84547) (Abcam, Cambridge, UK) for one hour at room temperature, then thoroughly washed three times with PBS. Bound antibodies were detected using the LSAB+ kit (Dako, Glostrup, Denmark). The slides were then counterstained with hematoxylin stain solution. Paraffin-embedded sections of human colon carcinoma and ovarian carcinoma of cytoplasmic immunoreactivity were used as positive controls. Negative controls had the primary antibody omitted and replaced by PBS. CIP2A immunoreactivity was scored as negative, weak, moderate, and strong expression, respectively.

This study was approved by the ethics committee of the Institutional Review Board of Taipei Veterans General Hospital. All informed consents from sample donors were in accordance with the Declaration of Helsinki and were obtained at time of their donation.

### Statistical analysis

Data are expressed as mean ± standard deviation (SD) or standard error (SE). Statistical comparisons were based on nonparametric tests and statistical significance was defined at *P *< 0.05. All statistical analyses were performed using SPSS for Windows software, version 12.0 (SPSS, Inc., Chicago, IL, USA).

## Results

### Differential apoptotic effects of bortezomib on breast cancer cells

**Figure 1:**
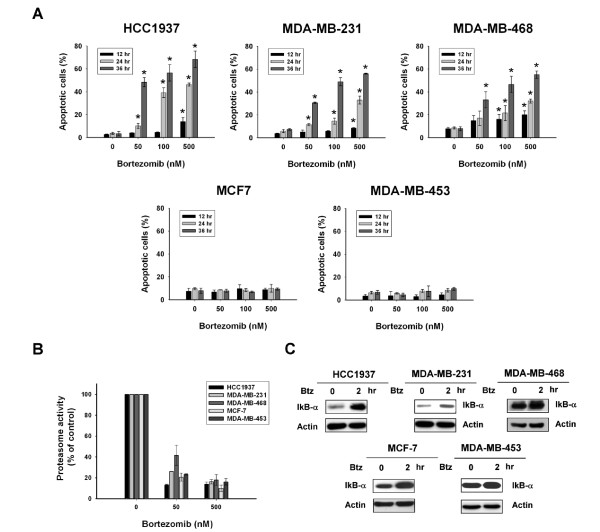
**Differential apoptotic effects and similar proteasome inhibition induced by bortezomib in breast cancer cells**. **A**, Dose- and time-escalation effects of bortezomib (50 nM, 100 nM and 500 nM) on apoptosis in five breast cancer cell lines (HCC-1937, MDA-MB-231, MDA-MB-468, MCF-7 and MDA-MD-453). Cells were exposed to bortezomib at the indicated doses for 12, 24 and 36 hours. Apoptotic cells were determined by flow cytometry. Columns, mean (*n *= 3); bars, SD; *P < 0.05. **B**, Bortezomib exerted an efficient and similar dose-dependent effect on proteasome inhibition as measured by proteasome activity assays in the five breast cancer cell lines. Cells were exposed to bortezomib at the indicated doses for 24 hours before measurement of proteasome activity. Columns, mean (*n *= 3); bars, SD; *P < 0.05. **C**, Bortezomib increased cytoplasmic protein levels of IκB-α, a cellular inhibitor of NF-κB and a known proteasome substrate, both in sensitive (HCC-1937, MDA-MB-231 and MDA-MB-468) and resistant (MCF-7 and MDA-MB-453) cells, suggesting similar proteasome inhibition in all these cells. Cells were treated with DMSO or 500 nM bortezomib at the indicated time point. Cytoplasmic extracts were prepared and assayed for IκB-α by western blot. Data are representative of three independent experiments. DMSO, dimethyl sulfoxide; SD, standard deviation.

To investigate the antitumor effect of bortezomib on breast cancer cells, we first assessed the apoptotic effect of bortezomib in a panel of five human breast cancer cell lines: TNBC cells HCC-1937, MDA-MB-231, and MDA-MB-468; HER2-overexpressing cells MDA-MB-453; and estrogen receptor positive cells MCF-7. Flow cytometry analysis of sub-G1 cells showed that bortezomib induced differential apoptotic effects at the indicated doses (50, 100 and 500 nM) on the five breast cancer cell lines (Figure 1A). Bortezomib induced apoptosis in a dose- and time-dependent manner in HCC-1937, MDA-MB-231, and MDA-MB-468 cells, whereas no apparent apoptotic effects were elicited by bortezomib in MCF-7 and MDA-MB-453 cells at doses up to 500 nM at 12, 24, and 36 hours of treatment (Figure 1A). These data indicate that triple negative breast cancer cell lines HCC-1937, MDA-MB-231, and MDA-MB-468 cells were sensitive to the cytotoxic activity of bortezomib, whereas MCF-7 and MDA-MB 453 cells were resistant.

### Bortezomib exerts similar, efficient proteasome inhibition in both sensitive and resistant breast cancer cells

To explore the mechanism by which bortezomib induces apoptosis in these breast cancer cell lines, we first examined the proteasome inhibitory effects of bortezomib in the cell lines. Proteasome activity was measured in the five cell lines after treatment for 24 hours with bortezomib. As shown in Figure 1B, bortezomib treatment resulted in similar dose-dependent effects on proteasome inhibition in all five breast cancer cell lines. Previous studies have shown that bortezomib inhibited NF-kB signaling through inhibiting the proteasome degradation of IkB (inhibitor of NF-kB) [4-6]. Therefore, we next examined the cytoplasmic protein levels of IkB in sensitive HCC-1937, MDA-MB-231, and MDA-MB-468 cells and resistant MCF-7 and MDA-MB-453 cells treated with bortezomib (Figure 1C). Notably, bortezomib increased the cytoplasmic protein levels of IκB-α both in sensitive and resistant cells, suggesting similar proteasome inhibition in all these cells. These results suggest that the differential induction of apoptosis by bortezomib in breast cancer cells may not necessarily be dependent on proteasome inhibition, which is consistent with our previous findings in HCC cells [17].

### Bortezomib induces apoptosis by downregulating CIP2A and p-Akt in sensitive TNBC cells

**Figure 2:**
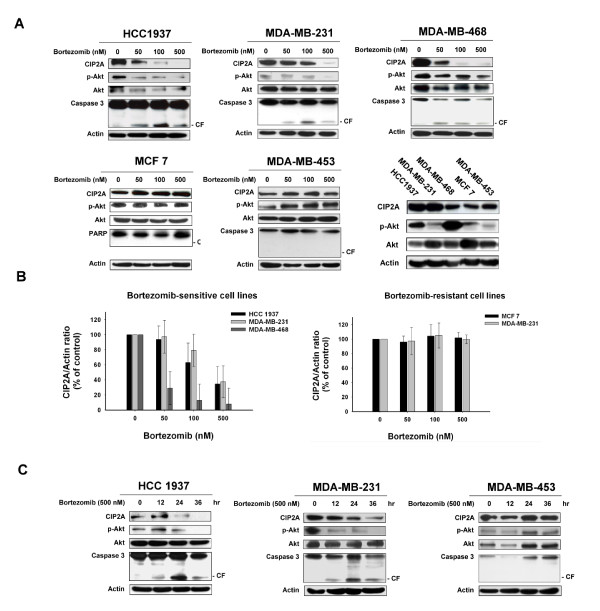
**Bortezomib downregulated CIP2A, p-Akt and induced cleavage of caspase 3 in a dose- and time-dependent manner**. **A**, Dose-dependent analysis of CIP2A, p-Akt and caspase-3. Cells were exposed to bortezomib at the indicated doses for 36 hours. Cell lysates were prepared and assayed for these molecules by western blotting. Data are representative of three independent experiments. CF, cleaved form (activated form). Note that PARP cleavage, instead of caspase-3, was used for assessment of apoptosis in MCF-7 cells since previous studies have shown that MCF-7 cells do not express caspase-3 due to the functional deletion of the *CASP*-3 gene [34-36]. Lower right, relative expression levels of CIP2A, p-Akt and Akt among all the cell lines tested are also shown. **B**, Ratio of CIP2A to actin levels from the cell lysates in (A). Immunoblots were scanned by a UVP BioSpectrum AC image system and quantitated using VisionWork LS software. Columns, mean (*n *= 3); bars, SD. **C**, Time-dependent analysis of CIP2A, p-Akt and caspase-3. Cells were exposed to bortezomib at 500 nM for the indicated time points. Data are representative of three independent experiments. CIP2A, cancerous inhibitor of PP2A; PARP, poly ADP-ribose polymerase; SD, standard deviation.

Our previous study demonstrated that the CIP2A-PP2A-AKT pathway mediated the sensitizing effect of bortezomib on tumor necrosis factor-related apoptosis-inducing ligand (TRAIL)-induced cell apoptosis in HCC [21]. Here we examined the molecular events associated with apoptosis and investigated the effects of bortezomib on p-Akt and CIP2A in bortezomib-treated breast cancer cells. As shown in Figure 2, bortezomib downregulated protein levels of CIP2A and induced apoptosis in sensitive HCC-1937, MDA-MB-231 and MDA-MB 468 cells in a dose-dependent manner (Figure 2A). Furthermore, inhibition of CIP2A was associated with downregulation of p-Akt and induction of apoptosis which was evidenced by the activation (cleavage) of caspase-3 in sensitive cells (Figure 2A). In contrast, bortezomib did not affect protein levels of CIP2A or P-Akt or induction of apoptosis in resistant MCF-7 and MDA-MB-453 cells (Figure 2A). It should be noted that we used PARP cleavage instead of caspase-3 for assessment of apoptotic events in the MCF-7 cell line because previous studies have shown that MCF-7 cells do not express caspase-3 due to the functional deletion of the *CASP*-3 gene [34-36]. In addition, relative expression levels of CIP2A, p-Akt and Akt among all the cell lines tested were also shown, and the intrinsic expression levels of these molecules seemed not to be correlated with bortezomib drug sensitivity (Figure 2A). Figure 2B shows the quantified ratio of CIP2A to actin levels as scanned from the cell lysates in Figure 2A and demonstrates the significant downregulation of CIP2A by bortezomib in sensitive cells in contrast with no significant alteration in CIP2A level in resistant cells. Similar effects of bortezomib on p-Akt and CIP2A can be seen in a time-dependent manner in bortezomib-treated breast cancer cells (Figure 2C). These results suggest that inhibition of CIP2A may be the major determinant of bortezomib-induced apoptosis in breast cancer cells.

### Target validation of CIP2A as a molecular determinant for bortezomib-induced apoptosis

**Figure 3:**
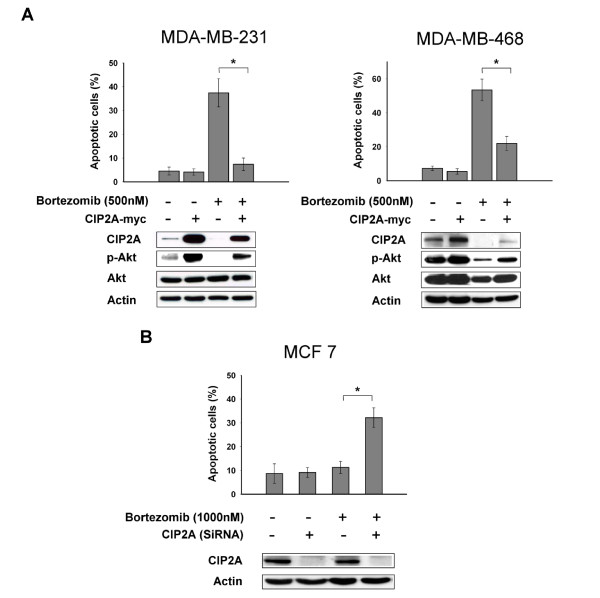
**Target validation of CIP2A**. **A**, Ectopic expression of CIP2A protected MDA-MB-231 (left) and MDA-MB-468 (right) cells from the apoptotic effect of bortezomib. Note that MDA-MB-231 and MDA-MB-468 cells with ectopic expression of CIP2A also had constitutively higher expression of p-Akt compared to wild-type cell clones. Columns, mean (*n *= 3); bars, SD; *P < 0.05. Cells were transfected with CIP2A and were selected for eight weeks by G-418. Analysis of apoptotic cells was performed by flow cytometry after cells were sequentially exposed to DMSO or bortezomib 500 nM for 36 hours. **B**, Downregulation of CIP2A by siRNA increased bortezomib-induced apoptosis in MCF-7 cells. Columns, mean (*n *= 3); bars, SD; *P < 0.05. Cells were transfected with either control (scrambled siRNA) or CIP2A siRNA for 48 h oursand then exposed to bortezomib at 1000 nM for 36 hours. CIP2A, cancerous inhibitor of PP2A; DMSO, dimethyl sulfoxide; SiRNA, small interfering RNA; SD, standard deviation.

To validate the role of CIP2A signaling in mediating the apoptotic effect of bortezomib in breast cancer cells, we first generated MDA-MB- 231-CIP2A and MDA-MB-468-CIP2A cells which constitutively express ectopic CIP2A (Figure 3A). Notably, MDA-MB-231-CIP2A and MDA-MB-468-CIP2A cells also expressed constitutively activated p-Akt, and constitutive ectopic expression of CIP2A protected sensitive MDA-MB 231 and MDA-MB 468 cells from apoptotic death induced by bortezomib (Figure 3A). Next, we knocked down protein expression of CIP2A in resistant MCF-7 cells by using siRNA. As shown in Figure 3B, downregulation of CIP2A sensitized resistant MCF-7 cells to bortezomib-induced apoptosis (Figure 3A). These results indicate that CIP2A plays a key role in mediating the apoptotic effect of bortezomib in breast cancer cells.

### Bortezomib downregulates transcription of CIP2A in breast cancer cells

**Figure 4:**
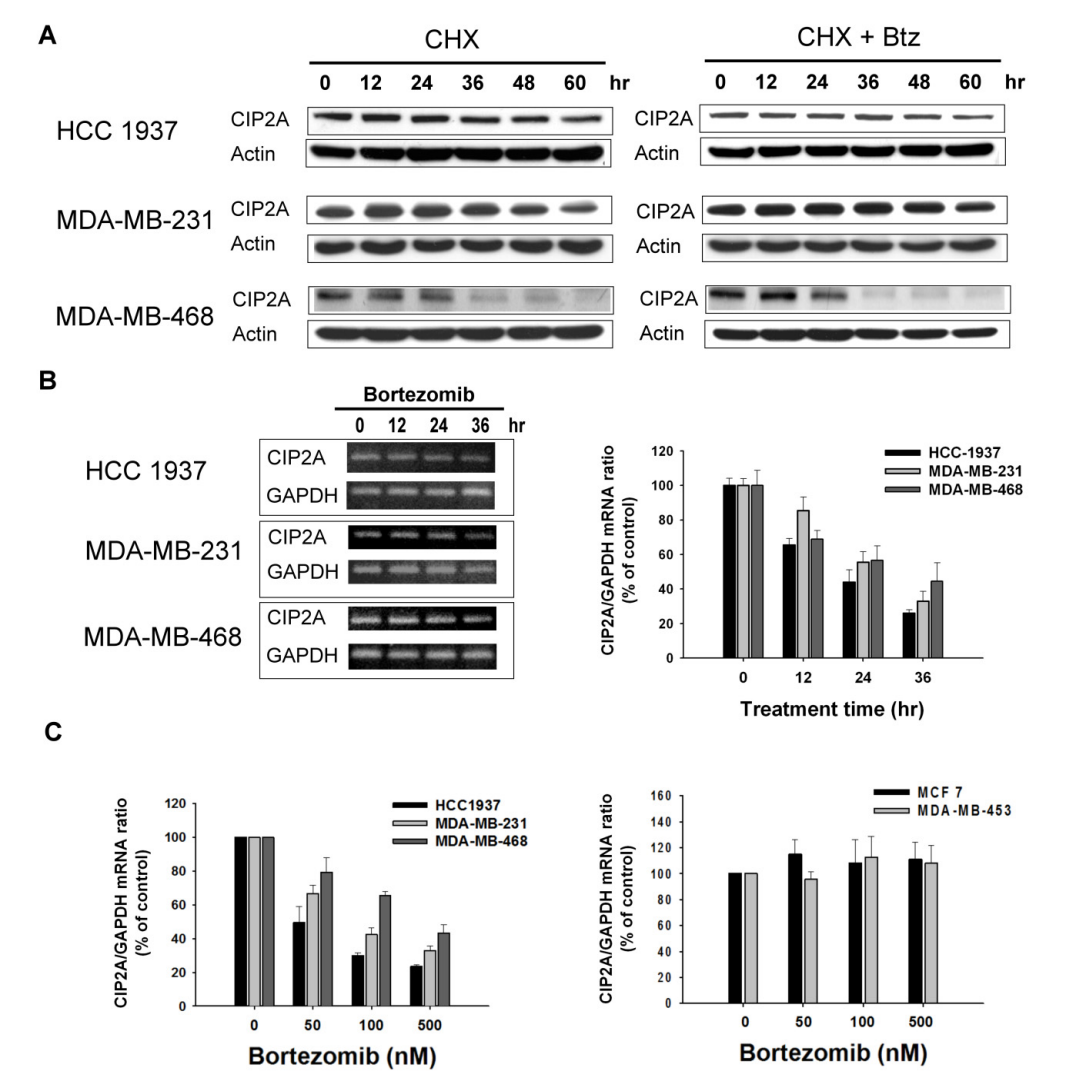
**Bortezomib downregulates transcription of CIP2A**. **A**, After cells were treated with 100 μg/ml translation inhibitor cyclohexamide (CHX) in the presence or absence of 500 nM bortezomib for the indicated length of time, the stability of CIP2A protein in whole-cell lysates were assessed by western blot. In bortezomib-sensitive cells (HCC 1937, MDA-MB-231, and MDA-MB-468), the addition of bortezomib did not affect CIP2A degradation. **B**, Bortezomib inhibits CIP2A mRNA in a time-dependent manner. Cells were treated with bortezomib at 500 nM for the indicated length of time, after which total RNA was isolated and CIP2A mRNA were semi-quantified using PCR as described in Methods. Columns, mean (*n *= 3); bars, SD. **C**, Bortezomib inhibits CIP2A mRNA in a dose-dependent manner in sensitive cells (left), but not in resistant cells (right). Cells were treated with bortezomib at the indicated doses for 36 hour, after which total RNA was isolated and CIP2A mRNA was analyzed by real-time quantitative PCR (qRT-PCR). Columns, mean (*n *= 3); bars, SD. CIP2A, cancerous inhibitor of PP2A; PCR, polymerase chain reaction; SD, standard deviation.

To examine the effects of bortezomib on CIP2A expression, we first examined whether bortezomib could affect CIP2A elimination (degradation) when translation was blocked by the protein synthesis inhibitor cycloheximide. Our data showed that after protein translation was blocked by cycloheximide, the rate of CIP2A degradation did not change significantly with or without bortezomib treatment in any of the three sensitive TNBC lines (HCC-1937, MDA-MB-231, and MDA-MB-468) (Figure 4A), suggesting that the effect of bortezomib on CIP2A may occur at the pre-translation level and that CIP2A might not be the proteasome degradation substrate. We next investigated whether bortezomib affected CIP2A transcription. As shown in Figure 4B, mRNA levels of CIP2A (as measured by a semi-quantitative nested PCR), decreased in a time-dependent manner upon treatment with bortezomib in the three sensitive breast cancer cell lines. Similarly, we performed real-time quantitative PCR analysis and showed that bortezomib inhibited mRNA levels of CIP2A in a dose-dependent manner in sensitive cell lines (Figure 4C, left) but not in resistant cell lines (Figure 4C, right). Failure of inhibiting CIP2A transcription by bortezomib suggests drug-resistance. Further studies are indicated to unravel the mechanisms underlying resistance to bortezomib-induced down regulation of CIP2A in MCF-7 and MDA-MB-453 cells.

### Effect of bortezomib on breast cancer xenograft tumor growth in vivo

**Figure 5:**
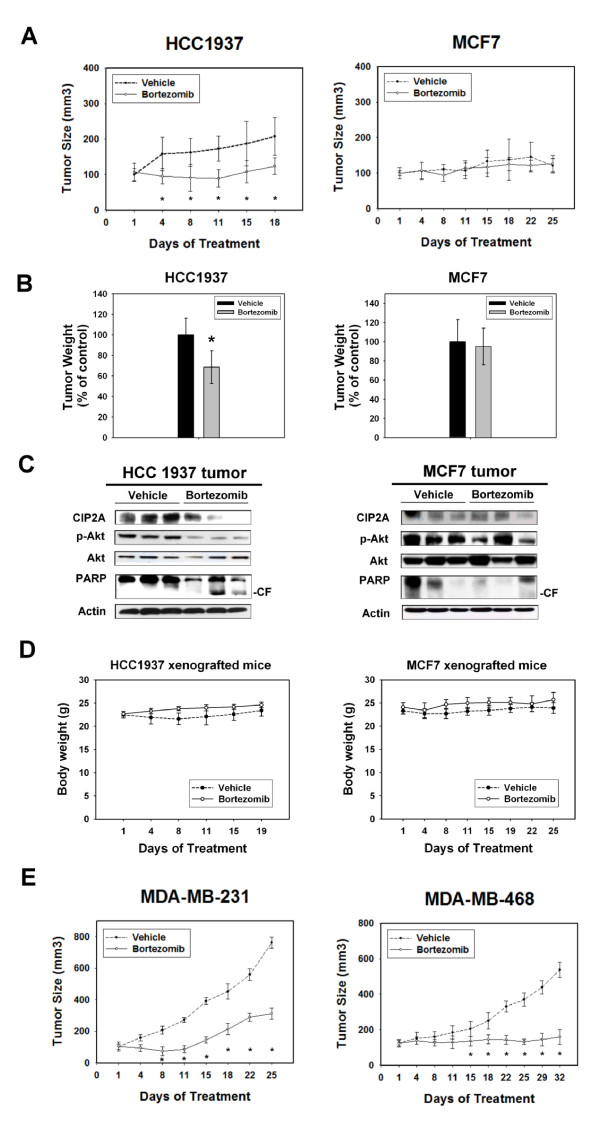
***In vivo *effect of bortezomib on human breast cancer cell lines xenograft nude mice**. **A**, Bortezomib decreased the size of HCC 1937 tumors (left), but had no anti-tumor effects on MCF-7 tumors (right). Points, mean (*n *= 6); bars, SE; * *P *< 0.05. B, Bortezomib reduced the weight of HCC 1937 tumors (left), but had no effect on that of MCF-7 tumors (right). Columns, mean (*n *= 6); bars, SD; * *P *< 0.05. **C**, Western blot analysis of CIP2A, p-Akt, Akt, and PARP cleavage in HCC 1937 and MCF-7 tumors. *In vivo *evidence of apoptosis is shown by PARP cleavage in HCC1937 tumors. ***D***, Body weight of xenograft mice bearing HCC 1937 tumors (left) and MCF-7 tumors (right) during the *in vivo *experiment. Points, mean (*n *= 6); bars, SE. **E**, Bortezomib inhibited tumor growth of another two triple negative breast tumor xenografts (MDA-MB-231 and MDA-MB-468). Points, mean (*n *= 6); bars, SE; * *P *< 0.05. Male NCr athymic nude mice (five to seven weeks of age) were used for experiments (A) to (D), and female athymic nude mice were used for experiment (E). Mice were treated with intra-peritoneal injections of bortezomib (1 mg/kg body weight) twice weekly. Controls received vehicle. CIP2A, cancerous inhibitor of PP2A; HCC, hepatocellular carcinoma; PARP, poly ADP-ribose polymerase; SD, standard deviation; SE, standard error.

To confirm that the effect of bortezomib on CIP2A has potentially relevant clinical implications in breast cancer, we assessed the *in vivo *effect of bortezomib on breast cancer xenograft tumors. HCC-1937 xenografted and MCF-7 xenografted tumor mice were generated to validate the role of CIP2A *in vivo*. Tumor-bearing mice were treated with vehicle or bortezomib i.p. at the clinically relevant dose of 1 mg/kg twice a week for three to four weeks. As shown in Figure 5, bortezomib significantly inhibited HCC-1937 tumor growth (*P *< 0.05) and the mean tumor size in the bortezomib treatment group was about 40% that of control at the end of treatment (Figure 5A, left). The mean tumor weight was also significantly reduced in bortezomib-treated mice (Figure 5B, left). In contrast, bortezomib did not inhibit MCF-7 tumor growth, as measured by tumor size (Figure 5A, right) and tumor weight (Figure 5B, right). To correlate the biological response with the mechanism of action identified *in vitro*, the effect of bortezomib on CIP2A in these tumors was examined by western blot. Figure 5C shows western blots of CIP2A, p-Akt, Akt, and PARP cleavage in the homogenates of three representative HCC-1937 and MCF-7 tumors. Overall, there was a significant decrease in CIP2A as well as p-Akt and evident apoptosis (as shown by PARP cleavage) in HCC-1937 tumors treated with bortezomib, whereas no significant change was observed in control (vehicle) or MCF-7 tumors (Figure 5C). Importantly, all animals tolerated the treatments well without observable signs of toxicity and had stable body weights throughout the course of the study (Figure 5D). No gross pathologic abnormalities were noted at necropsy. Finally, we examined whether bortezomib also inhibited other TNBC xenograft tumors *in vivo*. As shown in Figure 5E, bortezomib significantly inhibited MDA-MB-231 and MDA-MB-468 tumor growth *in vivo*.

### CIP2A expression in breast tumor tissue from patients with TNBC

**Figure 6:**
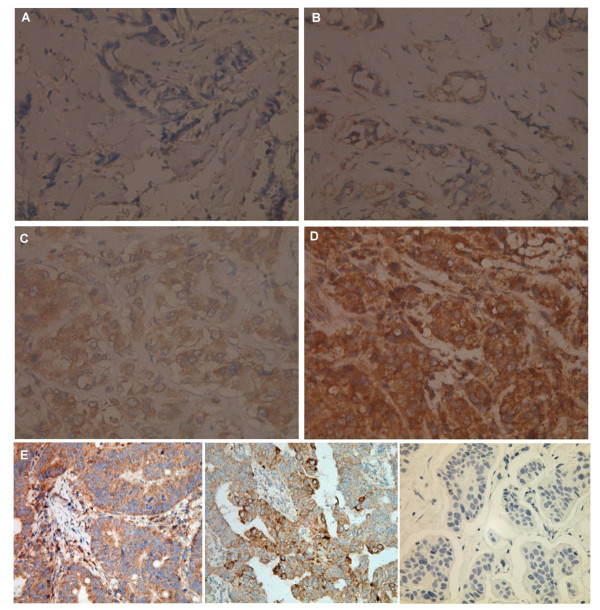
**Variable CIP2A expression in breast tumor tissue from patients with triple negative breast cancers**. Representative results of immunohistochemical staining for CIP2A in breast tumor tissue from patients with triple negative breast cancers, showing variable degree of CIP2A expression ranging from negative (A), weak (B), moderate (C), and strong (D) expression. E. Detection of human CIP2A cytoplasmic expression in colon carcinoma (left) and ovarian carcinoma (middle) by immunohistochemistry was used as positive control. Primary CIP2A antibody omitted and replaced by phosphate-buffered saline was performed as negative control in TNBC (right). (High power, 400X).

Previous study showing CIP2A expression in breast cancer has mainly been based on mRNA expression [33]. In the current study, we performed immunohistochemical (IHC) staining of CIP2A in a tissue array of 57 tumor samples from TNBC patients. Results showed that 50/57 (87.7%) tumors demonstrated variable CIP2A expression, among which 18(36%), 12(24%), and 20(40%) cases exhibited weak, moderate, and strong expression, respectively. Figure 6 demonstrates representative results of IHC staining for CIP2A, showing negative (A), weak expression (B), moderate expression (C), and strong expression (D). In contrast to the variable expression in cancer cells, normal stromal tissue in the adjacent areas of cancer did not stain with CIP2A (Figure 6A to 6D). Figure 6E shows a positive control from human colon carcinoma and ovarian carcinoma with cytoplasmic immunoreactivity (Figure 6E, left and middle) and a negative control which had the primary CIP2A antibody replaced by PBS (Figure 6E, right). Further studies as well as more samples are warranted to clarify the clinical role of CIP2A among various subtypes of breast cancer, in addition to TNBC subtypes.

## Discussion

This study reveals a novel mechanism by which bortezomib induces apoptosis in triple negative breast cancer cells (that is, CIP2A-dependent p-Akt downregulation). This finding has several potentially important implications: First, we identified CIP2A as a molecular determinant of cell sensitivity to bortezomib-induced apoptosis and demonstrated that bortezomib-induced apoptosis does not necessarily depend on its proteasome inhibition. We showed that bortezomib exerts similar proteasome inhibitory effects in sensitive and resistant cells as demonstrated by proteasome activity analysis and by efficient I-kB (a proteasome substrate) accumulation (Figure 1B and 1C). In contrast, bortezomib induced differential apoptotic effects in these breast cancer cells, which correlated with CIP2A downregulation (Figure 2 and 3). This *in vitro *finding was supported by previous *in vivo *evidence showing that bortezomib-induced proteasome inhibition did not correlate well with clinical therapeutic benefit in patients with breast cancers in a phase II study [12]. Indeed, despite the excellent anti-cancer activity of bortezomib in multiple myeloma and mantle cell lymphoma via its proteasome inhibition, cumulative clinical data have shown that bortezomib is less efficient or shows transient anti-cancer activity in solid tumors and other hematological malignancies [11,12,37-39]. Our data may, further, partly explain why bortezomib showed limited anti-tumor activity in breast cancer patients in the phase II trials [11,12]. In addition to the several known mechanisms of bortezomib resistance in cancers [4,40], the CIP2A-PP2A-p-Akt pathway may contribute to bortezomib resistance. Future studies correlating response to bortezomib with downregulation and/or pre-treatment expression levels of CIP2A in breast cancer patients may help to establish a clinical role for CIP2A as a predictive factor in breast cancer.

Second, our data strengthen the case for the use of CIP2A as an anti-cancer target. Accumulating evidence from our studies and the studies of others suggests that targeting CIP2A may be an ideal approach [26,32,33,41-43]. CIP2A expression is very low in most human tissues and, importantly, undetectable in normal mammary glands [32,33], thereby creating a potential therapeutic window for CIP2A targeting agents. Come *et al. *[33] demonstrated that depletion of CIP2A by siRNA inhibits tumor growth of MDA-MB-231 xenograft tumors. Our *in vivo *data also showed bortezomib downregulated CIP2A in HCC-1937 xenograft tumors and inhibited their tumor growth (Figure 5A to 5C). Moreover, it has been shown that the traditional chemotherapeutic agent doxorubicin downregulates CIP2A expression and that increased CIP2A expression confers doxorubicin resistance in breast cancer cells [44]. More recently, a natural Chinese medicinal herbal extract of *Rabdosia coetsa*, rabdocoetsin B, was also shown to inhibit proliferation and induce apoptosis in a variety of lung cancer cells via CIP2A-dependent p-Akt downregulation [43]. Taken together, these structurally unrelated agents show a common target in various cancer cells suggesting that CIP2A is a novel anti-cancer target.

Our data showed that 50/57 (87.7%) tumor samples from TNBC patients demonstrated variable CIP2A expressions. As stated earlier, CIP2A expression has been shown to correlate with disease aggressiveness [33] in breast cancer. Higher CIP2A expression has been shown as a prognostic factor predicting survival in gastric cancer [29], non-small cell lung cancer [27,45], renal cell carcinoma [46], serous ovarian cancer [47] and early stage tongue cancer [47]. Very recently, an IHC-based study [48] demonstrated that the CIP2A signature clustered with basal-type and HER2-positive breast cancer signatures and suggested that CIP2A is linked to these two subtypes of breast cancer. It would also be interesting to further investigate the prognostic role of CIP2A among various subtypes of breast cancer, in addition to TNBC subtypes, by large immunohistochemistry-based studies.

Despite the current results, the detailed mechanism by which bortezomib inhibits CIP2A remains unknown and further mechanistic studies are needed. Our data showed that bortezomib did not affect the half-life of CIP2A protein degradation after translation was stopped by cyclohexamide and that bortezomib suppressed CIP2A transcription (Figure 5), suggesting that the effect of bortezomib on CIP2A occurs pre-translation and is possibly irrelevant to its proteasome inhibition. The possible mechanisms through which bortezomib may affect the transcription of CIP2A include direct or indirect promoter regulation of CIP2A mRNA, epigenetic regulation of the *CIP2A *gene by DNA methylation or micro-RNA machinery, or affecting other uncovered molecules that regulate CIP2A expression.

## Conclusions

Bortezomib shows a favorable apoptosis-inducing effect in TNBC cells through a novel mechanism: CIP2A-dependent p-Akt downregulation. This study identified CIP2A as a major molecular determinant of the sensitivity of TNBC cells to bortezomib-induced apoptosis. This study also suggests that focusing on the interactions of oncoproteins, phosphatases and kinases could be a novel anti-cancer strategy. Future studies defining CIP2A as a useful therapeutic biomarker for breast cancer patients, as well as the detailed mechanism by which bortezomib inhibits CIP2A may lead to further progress in the development of molecular-targeted therapy for TNBC.

## Abbreviations

CIP2A: cancerous inhibitor of PP2A; (D)MEM: (Dulbecco's) modified Eagle's medium; DMSO: dimethyl sulfoxide; ER: estrogen receptor; FBS: fetal bovine serum; HCC: hepatocellular carcinoma; HER2: human epidermal growth factor receptor type 2; I-κB: inhibitor of NF-κB; IHC: immunohistochemical; i.p.: intraperitoneal; MBC: metastatic breast cancers; NF-κB: nuclear factor-κB; PARP: poly ADP-ribose polymerase; PBS: phosphate-buffered saline; PgR: progesterone receptor; PI3K: phosphatidylinositol-3-kinase; PP2A: protein phosphatase 2A; PTEN: phosphatase and tensin homologue deleted on chromosome ten; RT-PCR: reverse transcriptase polymerase chain reaction; s.c.: subcutaneous; siRNA: small interfering RNA; TNBC: triple negative breast cancer;.

## Competing interests

The authors declare that they have no competing interests.

## Authors' contributions

CYL and KFC were responsible for coordination and manuscript editing as well as acting as corresponding authors. CYL, KFC and CWS participated in the research design. LMT, KCC, CYL and PYC conducted experiments. LMT, CYL and CWS performed data analysis. LMT, CYL and KCC wrote or contributed to the writing of the manuscript. All authors have read and approved the final manuscript.
